# Deletions of the *Plasmodium falciparum* histidine-rich protein 2/3 genes are common in field isolates from north-eastern Tanzania

**DOI:** 10.1038/s41598-022-09878-3

**Published:** 2022-04-06

**Authors:** Robert D. Kaaya, Reginald A. Kavishe, Filemon F. Tenu, Johnson J. Matowo, Franklin W. Mosha, Chris Drakeley, Colin J. Sutherland, Khalid B. Beshir

**Affiliations:** 1 Kilimanjaro Christian Medical University College, Moshi, Tanzania; 2 Pan-African Malaria Vector Research Consortium, Moshi, Tanzania; 3 Department of Infection Biology, Faculty of Infectious & Tropical Diseases, London School of Hygiene and Tropical Medicine, London, UK

**Keywords:** Molecular biology, Biological techniques

## Abstract

*Plasmodium falciparum* parasites lacking *histidine-rich protein* 2 and 3 (*pfhrp2/3)* genes have been reported in several parts of the world. These deletions are known to compromise the effectiveness of HRP2-based malaria rapid diagnostic tests (HRP2-RDT). The National Malaria Control Programme (NMCP) in Tanzania adopted HRP2-RDTs as a routine tool for malaria diagnosis in 2009 replacing microscopy in many Health facilities. We investigated *pfhrp2/3* deletions in 122 samples from two areas with diverse malaria transmission intensities in Northeastern Tanzania. *Pfhrp2* deletion was confirmed in 1.6% of samples while *pfhrp3* deletion was confirmed in 50% of samples. We did not find parasites with both *pfhrp2* and *pfhrp3* deletions among our samples. Results from this study highlight the need for systematic surveillance of *pfhrp2/3* deletions in Tanzania to understand their prevalence and determine their impact on the performance of mRDT.

## Introduction

Malaria continues to be a health problem in Sub-Saharan Africa (SSA), where the 10 countries with the highest infection rates and deaths in the world are found^1^. A recent resurgence of the disease is evident in areas where a significant decline in malaria cases was previously observed and this inevitably calls for scrutiny of the malaria control interventions currently in use^1^, which comprise a number of strategies^1,2^. Artemisinin-based combination therapy (ACT) is the approved regimen for malaria treatment^3^. The World Health Organization (WHO) recommends for parasitological confirmation to be carried out before this treatment is provided^4^. Microscopy, although still regarded as the gold standard in malaria diagnosis, faces several operational challenges^5,6^. In the resource-limited settings of SSA, shortage of personnel with expertise, and long turn-around time of results from the laboratory have been identified as obstacles to the effective microscopic diagnosis of malaria, particularly in rural settings^7,8^.

Rapid diagnostic tests have proven to be reliable and sensitive enough to replace microscopy as a routine technique for malaria diagnosis in symptomatic patients^9^. The National Malaria Control Program (NMCP) in Tanzania rolled out malaria Rapid Diagnostic Tests (mRDT) in 2009, achieving diagnostic coverage of around 90% in public and private health facilities in 2014, with microscopy being used in the remaining 10% of facilities in the country^10^. Over 80% of mRDTs manufactured worldwide are sold or distributed in SSA. Tanzania Medicines and Medical Devices Authority (TMDA) approved five brands of mRDTs to be used in Tanzania^11^. Accredited Drug Dispensing Outlets (ADDOs) sells an average of 40 RDT units every week, most of them are HRP2-based and there are more than 6000 outlets in Tanzania mainland^12,13^. Rapid diagnostic tests have different detection thresholds, but sensitivity and specificity decrease when parasitaemia is below 200 parasites/µl^14,15^. Given the high detection threshold, mRDT might miss malaria parasites that are at low densities.

The majority of mRDTs distributed in the SSA are for *Plasmodium falciparum* detection^16^, mostly utilizing the histidine-rich protein (HRP2), as the antigenic marker. HRP2 is a 60–105 kD water-soluble protein secreted by *P. falciparum* trophozoites (asexual stage), encoded by the sub-telomeric *pfhrp2* locus on chromosome 8^17,18^. HRP2 is abundantly secreted and easily detected in the peripheral blood circulation of the host even when the parasite has sequestered in the microvasculature of the organs^19,20^, making the protein an important diagnostic target.

Studies in the last decade have reported the presence of *P. falciparum* parasites lacking both the loci encoding HRP2 and its isoform HRP3 (encoded by *pfhrp3*)*,* which has sufficient similarity to HRP2 and is recognised by the monoclonal antibodies used on the RDT test strips. *P. falciparum* parasites lacking the locus are thus not detected by HRP2-RDT. In the Amazon region of South America, these deletions have a reported prevalence of 28.6%, leading to recommendations to immediately stop using HRP2*-*RDTs^21^. Evidence from Asian countries also suggests the presence of *P. falciparum* lacking *pfhrp2*, including in India with a prevalence of 4%^22^ and China-Maynmar border with a prevalence of 5%^23^. Across Africa, Eritrea reported the highest prevalence of *pfhrp2* and *pfhrp3* deletions of 80.8% and 92.3%, respectively, which prompted a switch to non-HRP2-RDTs^24,25^. Studies elsewhere in East Africa also showed evidence of low proportions of *pfhrp2/3* gene deletions from field isolates^26–29^.

As Tanzania embarks on a malaria pre-elimination strategy, RDTs will play a crucial role in case detection, and assessment of their performance is vital. This study set out to assess the performance of HRP2-RDTs in two areas in Tanzania with different malaria transmission intensities.

## Results

### Malaria parasite detection

A total of 998 blood samples, 472 from Moshi and 526 from Handeni sites were collected and investigated for pfhrp2/3 deletions using LDH/HRP2-RDT, microscopy and nested PCR. In Moshi site, only 1 sample (0.2%) was positive by mRDT while in the Handeni site, 203 (38.6%) samples were positive. Analysis by nested PCR revealed that 135 (13.5%) samples were positive, of which 19 (4%) positive samples were from Moshi and 116 (22%) positives were from Handeni as shown in Table 1.

**Table 1:** Malaria prevalence in the Handeni and Moshi district.

	Handeni				Moshi				Both Sites			
	N	%	95% C.I.		N	%	95% C.I.		N	%	95% C.I.	
			Lower	Upper			Lower	Upper			Lower	Upper
RTD positive*	203	38.59	35.42	43.88	1	0.21	0.03	1.49	204	20.44	18.29	23.36
Microscope positive	89	16.95	13.95	20.37	6	1.27	0.57	2.80	95	9.52	7.85	11.50
PCR positive	116	22.05	18.71	25.80	19	4.03	2.58	6.23	135	13.53	11.54	15.80

*HRP2 only or both HRP2 and LDH.

### Confirming *pfhrp2/3* deletion

**Figure 1:**
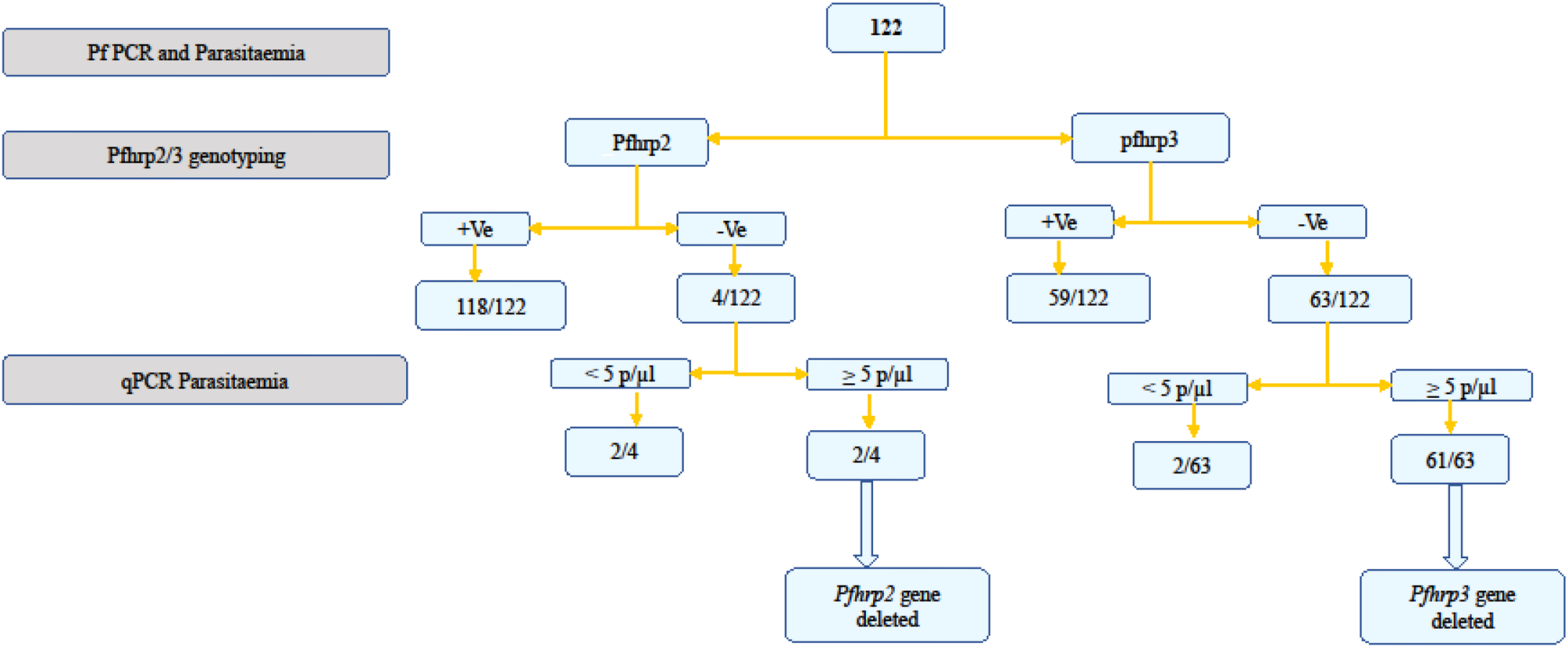
A flow chart showing analysis for determining *pfhrp2/pfhrp3* gene deletion.

A total of 122 samples that were positive for *P. falciparum* species-specific PCR or microscopy were analysed for *pfhrp2* and *pfhrp3* deletions. Of the 122 samples, four samples were *pfhrp2* PCR negative, and two of the four samples (1.6%) had relative parasitaemia of ≥ 5 p/µl and were considered true *pfhrp2* deletion. The two samples with the confirmed *pfhrp2* deletion had parasiteamia of 144 p/µl and 440 p/µl. *Pfhrp3* analysis showed that 52% (63/122) of the samples were negative, and further analysis on parasitaemia revealed that the majority (50%, n = 61 ) had a relative parasite density of ≥ 5/µl (Fig. 1). The parasitaemia of the *pfhrp3*-deleted samples ranges between 12 p/µl and 14,696 p/µl, with a mean parasite density of 1473 p/µl. The *pgmet, a* single-copy parasite gene, was used both for confirmation of DNA quality and to estimate parasite density.

### Effect of *pfhrp2/3* gene deletion on RDT performance

Of the 122 samples screened for *pfhrp2/3* deletion, four samples carried *pfhrp2*-deleted parasites and gave positive RDT signal; of which two samples reacted on HRP2 line only. On the other hand, only 2 out of 63 samples with *pfhrp3* deletion were RDT negative (Table 2). Overall, there were 32 samples with RDT negative results but *pfhrp2/3* positive had a mean parasitaemia of 14 p/µl, ranging between 1 p/µl and 142 p/µl.

**Table 2:** Discordance between microscopy, nPCR, RDT and pfhrp2/3 PCR results.

N = 122	nPCR + or Micro + &RDT − n (%)	nPCR + or Micro + &RDT + n (%)
Pfhrp2−	0 (0%)	4 (3%)
Pfhrp2+	16 (13%)	102 (84%)
Pfhrp3−	2 (1.6%)	61 (50%)
Pfhrp3+	14 (11.4%)	45 (37%)

### Impact of parasitaemia on *pfhrp2/3* gene detection

**Figure 2:**
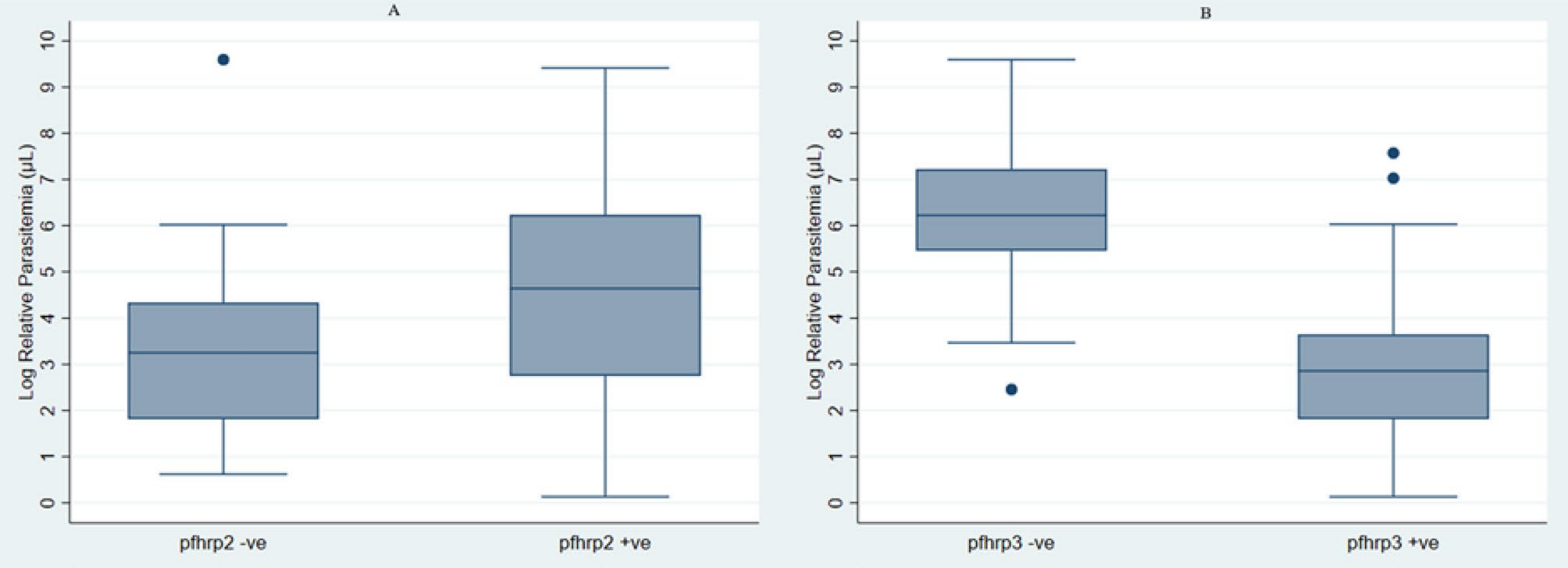
Parasitemia levels for *pfhrp-2/3* gene negative and positive. *log back-transformation was done on median values to obtain actual parasitaemia.

No statistically significant difference in parasitaemia was observed between the two *pfhrp2/3* groups. Median parasitaemia was 104 p/µl in the *pfhrp2* positive and 26 p/µl in the *pfhrp2*-deleted samples respectively (Fig. 2A). On the other hand, *pfhrp3* positive and *pfhrp3*-deleted samples had a median parasitaemia of 504 p/µl and 18 p/µl, respectively (Fig. 2B).

## Discussion

In this study, we report evidence of *pfhrp2/3* deletions in north-eastern Tanzania. We confirmed the presence of two samples with *pfhrp2* deletion, though they did not cause RDT negative result. This finding is also consistent with previous studies on samples from Tanzania and Yemen, where they found *pfhrp2* deletion on an mRDT positive sample^30,31^. This anomaly might be due to false-positive results on mRDT caused by cross-reaction with circulating proteins such as rheumatoid factor^32^ or the positive RDT signal may have arisen from the previous infection with *pfhrp2*-positive samples^33^. Our results also show a high proportion of *pfhrp3* deletion compared to *pfhrp2*. This finding is interesting, given *pfhrp3* deletion is suggested to be more common in low transmission season with minimal chance of polyclonal infection. Reports from Central and Southern America, where malaria transmission is low, showed similar observations, whereby up to 70% of the tested samples had a deletion on *pfhrp3* region^21,34,35^. Double deletions (*pfhrp2* + *pfhrp3*) were not observed in this study.

Guidelines on *pfhrp2/3* deletions rely solely on discordant results between microscopy and mRDT or a dual-antigen HRP2-RDT as an algorithm for suspecting the deletions^36^. All the microscopy positive samples were screened in this analysis, irrespective of symptoms, even if they had positive mRDT results. Studies have shown persistence of HRP2 in the plasma up to 28 days after treatment particularly in high parasitaemia infections^33^, in that case, a person can have a new infection with *P. falciparum* parasites lacking *pfhrp2/3* genes but have a circulating HRP2 from the previous infection commonly observed in areas with high malaria transmission. These findings suggest the importance of timing of the surveillance and considerations should be given to find optimum time when to survey *pfhrp2/3* deletions during the transmission season. In the absence of the *pfhrp2* locus, circulating HRP3 protein alone can give a positive HRP-RDT signal when parasitaemia is more than 1000 parasite/µl^37^. High sequence and structural homology between the two isoform proteins is the accepted reason for this^29,38,39^. This phenomenon can mask the deletion effect on either of the genes on the performance of mRDT at moderate to high parasite density.

The presence of *pfhrp2/3* deletions in asymptomatic individuals highlights the importance of surveying individuals with different disease spectrum and documenting the importance pfhrp2/3 deletion in disease outcome^40^. The WHO master protocol for surveillance of *pfhrp2/pfhrp3* deletion, which emphasizes a health-center-based approach targeting symptomatic individuals, could miss deletions in asymptomatic individuals^41^. However, it is not clear what proportion of the deletions is contributed from asymptomatic individuals, and their inclusion may not be operationally feasible. The use of HRP2-based RDT will likely exert selective pressure on the parasite population and could lead to the spread of *pfhrp2/3*-deleted parasites^42^.

## Conclusion

This study provides evidence of *pfhrp2* and *pfhrp3* deletions in *P. falciparum* isolates from Tanzania. This makes it urgent for systematic surveillance of *pfhrp2/3* deletions to understand the prevalence and extent of such deletions in Tanzania. The high proportion of *pfhrp3* deletion attracts attention and there is now a need to understand what drives these deletions through the transmission season. Findings from this study support the idea of screening for *pfhrp2/3* deletions even in mRDT positive samples, bearing in mind the cross-reactivity between the two proteins but also the false positivity of mRDTs due to persistence of plasma HRP2/HRP3 after treatment.

### Study limitations

The study was conducted in the middle of peak transmission season (April–June 2018) and in an area previously reported to have high polyclonal *P. falciparum* infections^43,44^. We might have underestimated gene deletions since we did not estimate the multiplicity of infection in the study area. A positive signal from non-falciparum parasites may have also caused a reaction in the LDH line but we couldn’t verify this as we did not have DNA left. The use of a high throughput multiplex qPCR targeting single copy parasite gene can resolve the veiled effect of polyclonal infection in *pfhrp2/3* gene deletion^39^.

## Methods

### Study area and participants

**Figure 3:**
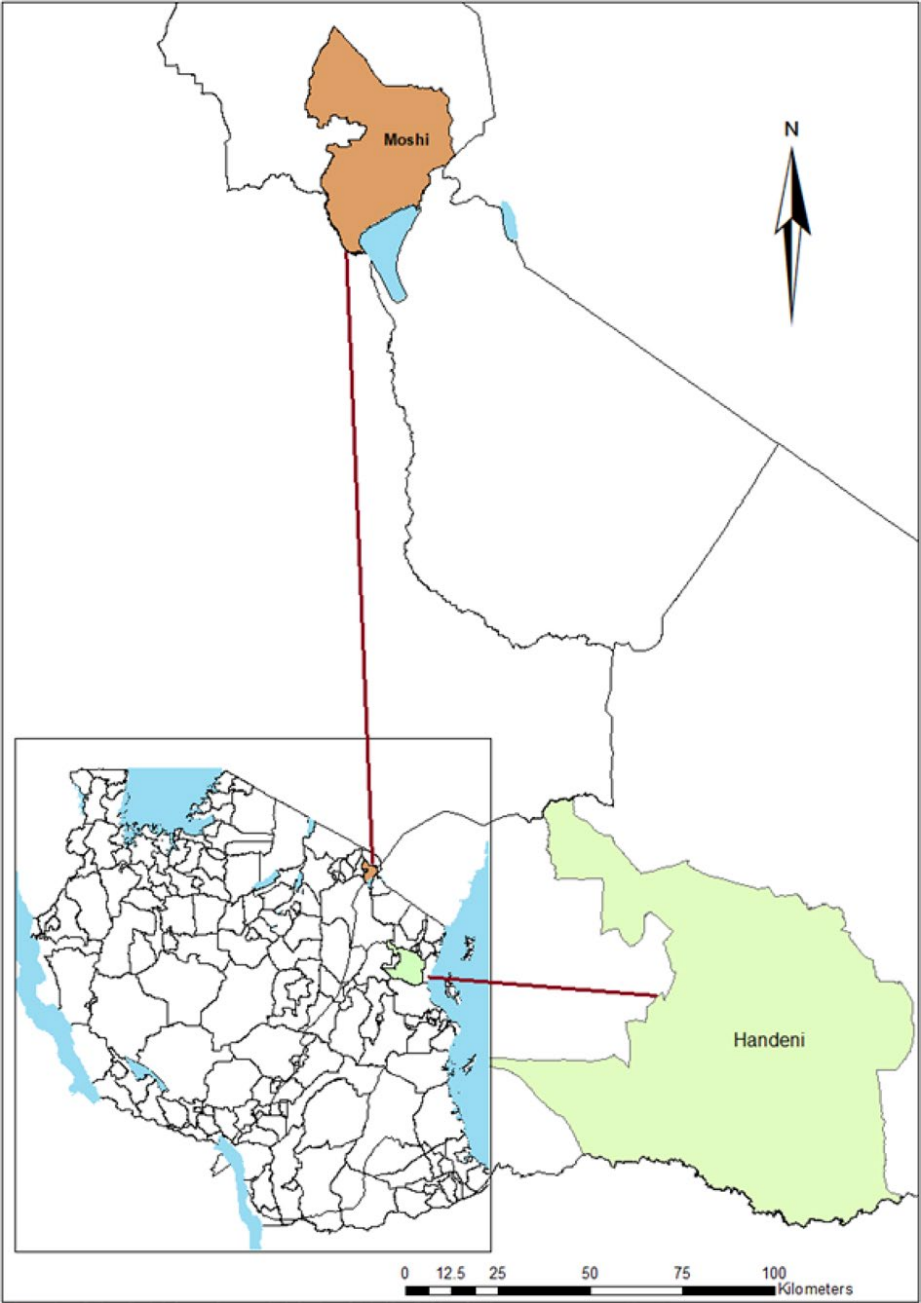
A map of Tanzania showing the study areas (map created using ArcGIS software v10.3).

This study was conducted in Lower-Moshi in Kilimanjaro and Handeni Tanga region (Fig. 3). Handeni is in the Tanga region, on the North-Eastern coast of Tanzania. The region is endemic for malaria with a perennial transmission pattern and is known to be a focal area for malaria drug resistance. It has an EIR of about ~ 100 infectious bites per person per year and a perennial prevalence of 25–30%^44–46^. The study area has two rainy seasons per year, which denotes the peaks of malaria transmission. The long rainy season is from March-June and the short rainy season is from October–November. The area is located at 309 m above sea level, 5°22′60″ N and 38°34′60″ E.

The second study site was Lower Moshi (3021′ S, 37020′ E), The area is about 800 m above sea level, south of Mount Kilimanjaro. It is one of the sentinel sites for insecticide resistance surveillance under the National Malaria Control Programme. Transmission of malaria in Lower Moshi occurs throughout the year with a prevalence of < 0.1%^47^.

### Sample size calculation

The sample size was calculated assuming the following parameters, proportion for *pfhrp2/3* gene deletion PCR is 0.5 (proportion in the population), Power = 0.80, Alpha = 0.05 (two-sided), Anticipated difference = 0.1, Alternative p = 0.4, Design effect = 2. The estimated required sample size in each site was 194*2 = 388 per study site, (Using STATA software). The total estimated minimum sample size was 776 in the two study sites. In this study we enrolled 1013 participants but presented results for only 998 participants, enrolment forms for 15 individuals had missing information and were dropped from the analysis.

### Participant recruitment

Community sensitization meetings were organized, whereby the study personnel explained the study and answered any questions in an open forum before participant recruitment. Enrolment occurred in health facilities in each site, participants were enrolled only after verification of potential eligibility, explaining the study in Swahili (local language) and consent to participate. Each member of the community had an equal chance of being selected as a participant.

### Sample collection and Malaria rapid diagnosis

Whole blood was collected just after the rainy season, from April to June 2018. The diagnosis on enrolled participants was done on-site, whereby a qualitative SD BIOLINE Malaria Ag P.f/Pan test (Standard diagnostics INC. Korea) detects histidine-rich protein II (HRP-II) antigen of *Plasmodium falciparum* and *Plasmodium* lactate dehydrogenase (pLDH) for the *Plasmodium* species.

### Blood smear microscopy

Thick and thin smears were prepared and left to air dry at room temperature, thin smears were carefully sprayed with absolute methanol for fixation. Staining was done with 10% Giemsa stain for 30 min, thereafter the stain was washed away by tap water and left to dry. Double reading of the slides was done by a separate microscopist and discordant results were then resolved by a third reader and results were presented as the number of parasites in every 200 white blood cells. Microscopists received their training from the Malaria Diagnostic Centre of Excellence in Kisumu Kenya^48^.

### DNA extraction

Genomic DNA was extracted from dried blood spots (DBS) using a robotic DNA extraction system (Qiasymphony, QIAGEN, Germany) at the London School of Hygiene Tropical Medicine (LSHTM)- UK, the method previously validated and published^49,50^.

### PCR confirmation of *Plasmodium falciparum* DNA

*Plasmodium falciparum* was detected using a standard nested-PCR technique targeting 18S ribosomal RNA, primers and PCR conditions were as previously published^51^.

### Estimation of parasite density

A qPCR that amplifies tRNA methionine of the *Plasmodium (PgMET)* gene and human beta-tubulin gene (*HumTUBB)* was used to relatively estimate parasite density in the samples. HumTUBB was used as an internal DNA extraction control and *Plasmodium falciparum* international standard as a calibrator^29,52^. Primers and probes used (Table 3), master mixes, and amplification profiles were adopted from Beshir et al. (2010)^53^.

**Table 3:** Primers and probes for parasite density qPCR.

Primers/Probes	5′ Modification	Sequence	3′ Modification
PgMET_F		5′-TGAAAGCAGCGTAGCTCAGA	
PgMET_R		5′-CGCGTGGTTTCGATCCACG	
PgMET_pr	FAM	5′-GGGGCTCATAACCCCCAGGA	BHQ2
HumTuBB_F		5′-AAGGAGGTCGATGAGCAGAT	
HumTuBB_R		5′-GCTGTCTTGACATTGTTGGG	
HumTuBB_pr	JOE	5′-TTAACGTGCAGAACAAGAACAGCAGCT	BHQ2

### Pfhrp2/Pfhrp3 genotyping

The amplification of *pfhrp2/3* genes from DBS samples was done using nested-PCR as described previously^21,54^. Primers used for nest-1 PCR were Pfhrp2-F1 (5′-CAAAAGGACTTAATTTAAATAAGAG-3′) and Pfhrp2-R1 (5′-AATAAATTTAATGGCGTAGGCA-3′). Nest-2 primers used were Pfhrp2-F2 (5′-ATTATTACACGAAACTCAAGCAC-3′) and Pfhrp2-R1. PCR reaction mix contained a final concentration of 0.2 µM for each primer, 0.5 µM of deoxynucleoside triphosphate (dNTP) mix (Promega), 0.2 U of Taq-polymerase (AmpliTaq Gold-Applied biosystems), and 5 µl of the DNA template. Amplification thermo-profile for the reaction was 95 °C for 10 min (Enzyme activation) followed by 40 cycles of 94 °C for 50 s, 50 °C for 30 s and 65 °C for 1 min. Final elongation was done at 70 °C for 15 min then at 4 °C until removed from the machine. Primers used for the amplification of Pfhrp3 gene were Pfhrp3-F1 (5′-AATGCAAAAGGACTTAATTC-3′), Pfhrp3-R1 (5′-TGGTGTAAGTGATGCGTAGT-3′), Pfhrp3-F2 (5′-AAATAAGAGATTATTACACGAAAG-3′) and Pfhrp3-R1. Master mix and amplification profiles were the same as for Pfhrp2. Laboratory strains Dd2 and HB3 were used as positive controls for *pfhrp2* and *pfhrp3* deletion respectively.

### Data analysis

A descriptive analysis was performed using Stata 16 software (StataCorp LLC, TX, USA), with proportions and frequencies detailed in tables and figures. A log transformation of relative parasitaemia was applied to compare the mean and median values. Box plots were used to show the median, the 25th, and 75th percentiles of the non-parametric test.

### Ethical approval

This study was assessed and approved by the Kilimanjaro Christian Medical University College Research Ethics Review Committee and given ethical clearance certificate # 2238 of the research proposal # 1084. Consent from study participants was sought before enrolment, guidelines and regulations that safeguard participants were also observed.

## Data Availability

The data sets developed during this investigation are not publicly available, however, they are available upon reasonable request from the corresponding author.

## References

[1] WHO. Global Malaria report, https://www.who.int/publications/i/item/9789240015791. (2020).

[2] WHO. World Malaria Report, https://www.who.int/malaria/publications/atoz/9789241564106/en/. (2010).

[3] Shibeshi W, Alemkere G, Mulu A, Engidawork E (2021). Efficacy and safety of artemisinin-based combination therapies for the treatment of uncomplicated malaria in pediatrics: A systematic review and meta-analysis. BMC Infect. Dis..

[4] WHO. Guidelines for the treatment of malaria, https://www.afro.who.int/publications/guidelines-treatment-malaria-third-edition. (2015).

[5] Falade CO (2016). Malaria rapid diagnostic tests and malaria microscopy for guiding malaria treatment of uncomplicated fevers in Nigeria and prereferral cases in 3 African countries. Clin. Infect. Dis..

[6] Mukry SN (2017). Laboratory diagnosis of malaria: Comparison of manual and automated diagnostic tests. Can. J. Infect. Dis. Med. Microbiol..

[7] Ashraf S (2012). Developing standards for malaria microscopy: External competency assessment for malaria microscopists in the Asia-Pacific. Malar. J..

[8] Birhanie M (2016). Comparison of partec rapid malaria test with conventional light microscopy for diagnosis of malaria in Northwest Ethiopia. J. Parasitol. Res..

[9] Cunningham J (2019). A review of the WHO malaria rapid diagnostic test product testing programme (2008–2018): Performance, procurement and policy. Malar. J..

[10] PMI. Tanzania Malaria operation plan, https://www.pmi.gov/where-we-work/tanzania. (2015).

[11] Ishengoma DS (2016). The role of malaria rapid diagnostic tests in screening of patients to be enrolled in clinical trials in low malaria transmission settings. Heal. Syst. Policy Res..

[12] Poyer S (2015). Availability and price of malaria rapid diagnostic tests in the public and private health sectors in 2011: Results from 10 nationally representative cross-sectional retail surveys. Trop. Med. Int. Heal..

[13] Maloney K (2017). Expanding access to parasite-based malaria diagnosis through retail drug shops in Tanzania: Evidence from a randomized trial and implications for treatment. Malar. J..

[14] Maltha J (2010). Evaluation of a rapid diagnostic test (CareStart™ Malaria HRP-2/pLDH (Pf/pan) Combo Test) for the diagnosis of malaria in a reference setting. Malar. J..

[15] Mouatcho JC, Goldring JPD (2013). Malaria rapid diagnostic tests: Challenges and prospects. J. Med. Microbiol..

[16] WHO. Malaria rapid diagnostic test performance. Results of WHO product testing of malaria RDTs: round 8. 172 (2018).

[17] Akinyi S (2013). Multiple genetic origins of histidine-rich protein 2 gene deletion in *Plasmodium falciparum* parasites from Peru. Sci. Rep..

[18] Sepúlveda N (2018). Global analysis of *Plasmodium falciparum* histidine-rich protein-2 (pfhrp2) and pfhrp3 gene deletions using whole-genome sequencing data and meta-analysis. Infect. Genet. Evol. J. Mol. Epidemiol. Evol. Genet. Infect. Dis..

[19] Marquart L, Butterworth A, McCarthy JS, Gatton ML (2012). Modelling the dynamics of *Plasmodium falciparum* histidine-rich protein 2 in human malaria to better understand malaria rapid diagnostic test performance. Malar. J..

[20] Ramutton T (2012). Sequence variation does not confound the measurement of plasma PfHRP2 concentration in African children presenting with severe malaria. Malar. J..

[21] Gamboa D (2010). A large proportion of *P. falciparum* isolates in the Amazon region of Peru lack pfhrp2 and pfhrp3: Implications for malaria rapid diagnostic tests. PLoS ONE.

[22] Kumar N (2013). Genetic deletion of HRP2 and HRP3 in Indian *Plasmodium falciparum* population and false negative malaria rapid diagnostic test. Acta Trop..

[23] Li P (2015). Genetic diversity of *Plasmodium falciparum* histidine-rich protein 2 in the China-Myanmar border area. Acta Trop..

[24] Berhane A (2018). Major threat to malaria control programs by *Plasmodium falciparum* lacking histidine-rich protein 2, eritrea. Emerg. Infect. Dis..

[25] Agaba BB (2019). Systematic review of the status of pfhrp2 and pfhrp3 gene deletion, approaches and methods used for its estimation and reporting in *Plasmodium falciparum* populations in Africa: Review of published studies 2010–2019. Malar. J..

[26] Parr JB (2017). Pfhrp2-deleted *Plasmodium falciparum* parasites in the Democratic Republic of the Congo: A national cross-sectional survey. J. Infect. Dis..

[27] Kozycki CT (2017). False-negative malaria rapid diagnostic tests in Rwanda: Impact of *Plasmodium falciparum* isolates lacking hrp2 and declining malaria transmission. Malar. J..

[28] Bosco AB (2020). Molecular surveillance reveals the presence of pfhrp2 and pfhrp3 gene deletions in *Plasmodium falciparum* parasite populations in Uganda, 2017–2019. Malar. J..

[29] Beshir KB (2017). *Plasmodium falciparum* parasites with histidine-rich protein 2 (pfhrp2) and pfhrp3 gene deletions in two endemic regions of Kenya. Sci. Rep..

[30] Thomson R (2019). pfhrp2 and pfhrp3 gene deletions that affect malaria rapid diagnostic tests for *Plasmodium falciparum*: Analysis of archived blood samples from 3 African countries. J. Infect. Dis..

[31] Atroosh WM (2015). Genetic variation of pfhrp2 in *Plasmodium falciparum* isolates from Yemen and the performance of HRP2-based malaria rapid diagnostic test. Parasit. Vectors.

[32] Gatton ML (2018). An assessment of false positive rates for malaria rapid diagnostic tests caused by non-Plasmodium infectious agents and immunological factors. PLoS ONE.

[33] Houzé S, Boly MD, Le Bras J, Deloron P, Faucher J-F (2009). PfHRP2 and PfLDH antigen detection for monitoring the efficacy of artemisinin-based combination therapy (ACT) in the treatment of uncomplicated falciparum malaria. Malar. J..

[34] Abdallah JF (2015). Prevalence of pfhrp2 and pfhrp3 gene deletions in Puerto Lempira, Honduras. Malar. J..

[35] Murillo Solano C (2015). Deletion of *Plasmodium falciparum* histidine-rich protein 2 (pfhrp2) and histidine-rich protein 3 (pfhrp3) genes in Colombian Parasites. PLoS ONE.

[36] WHO. Response plan to pfhrp2 gene deletions, https://apps.who.int/iris/bitstream/handle/10665/325528/WHO-CDS-GMP-2019.02-eng.pdf. (2019).

[37] Kong A (2021). HRP2 and HRP3 cross-reactivity and implications for HRP2-based RDT use in regions with *Plasmodium falciparum* hrp2 gene deletions. Malar. J..

[38] Wellems TE (1987). A histidine-rich protein gene marks a linkage group favored strongly in a genetic cross of *Plasmodium falciparum*. Cell.

[39] Grignard L (2020). A novel multiplex qPCR assay for detection of *Plasmodium falciparum* with histidine-rich protein 2 and 3 (pfhrp2 and pfhrp3) deletions in polyclonal infections. EBioMedicine.

[40] Nolder D (2021). Failure of rapid diagnostic tests in Plasmodium falciparum malaria cases among travelers to the UK and Ireland: Identification and characterisation of the parasites. Int. J. Infect. Dis..

[41] Organization WH (2020). Master Protocol for Surveillance of pfhrp2/3 Deletions and Biobanking to Support Future Research.

[42] Watson OJ (2017). Modelling the drivers of the spread of *Plasmodium falciparum* hrp2 gene deletions in sub-Saharan Africa. Elife.

[43] Kidima W, Nkwengulila G (2015). *Plasmodium falciparum msp2* genotypes and multiplicity of infections among children under five years with uncomplicated malaria in Kibaha. Tanzania. J. Parasitol. Res..

[44] Gesase S (2009). High resistance of *Plasmodium falciparum* to sulphadoxine/pyrimethamine in Northern Tanzania and the emergence of dhps resistance mutation at Codon 581. PLoS ONE.

[45] Alifrangis M (2009). Five-year surveillance of molecular markers of *Plasmodium falciparum* antimalarial drug resistance in Korogwe District, Tanzania: Accumulation of the 581G mutation in the *P. falciparum* dihydropteroate synthase gene. Am. J. Trop. Med. Hyg..

[46] Thomsen TT (2011). Prevalence of single nucleotide polymorphisms in the *Plasmodium falciparum* multidrug resistance gene (Pfmdr-1) in Korogwe District in Tanzania before and after introduction of artemisinin-based combination therapy. Am. J. Trop. Med. Hyg..

[47] Shekalaghe SA (2007). Submicroscopic *Plasmodium falciparum* gametocyte carriage is common in an area of low and seasonal transmission in Tanzania. Trop. Med. Int. Heal..

[48] Ohrt C (2007). Establishing a malaria diagnostics centre of excellence in Kisumu, Kenya. Malar. J..

[49] Kruhøffer M (2010). Evaluation of the QIAsymphony SP workstation for magnetic particle—Based nucleic acid purification from different Sample types for demanding downstream applications. J. Lab. Autom..

[50] Pillet S, Bourlet T, Pozzetto B (2012). Comparative evaluation of the QIAsymphony RGQ system with the easyMAG/R-gene combination for the quantitation of cytomegalovirus DNA load in whole blood. Virol. J..

[51] Snounou G, Singh B (2002). Nested PCR analysis of Plasmodium parasites. Methods Mol. Med..

[52] Robinson A (2019). Molecular quantification of Plasmodium parasite density from the blood retained in used RDTs. Sci. Rep..

[53] Beshir KB (2010). Measuring the efficacy of anti-malarial drugs in vivo: Quantitative PCR measurement of parasite clearance. Malar. J..

[54] Baker J (2005). Genetic diversity of *Plasmodium falciparum* histidine-rich protein 2 (PfHRP2) and its effect on the performance of PfHRP2-based rapid diagnostic tests. J. Infect. Dis..

